# HSP-90/kinase complexes are stabilized by the large PPIase FKB-6

**DOI:** 10.1038/s41598-021-91667-5

**Published:** 2021-06-11

**Authors:** Siyuan Sima, Katalin Barkovits, Katrin Marcus, Lukas Schmauder, Stephan M. Hacker, Nils Hellwig, Nina Morgner, Klaus Richter

**Affiliations:** 1 Department of Chemistry, Center for Integrated Protein Research, Technische Universität München, Lichtenbergstr. 4, 85748 Garching, Germany; 2 Medizinisches Proteom-Center, Ruhr-Universität Bochum, Gesundheitscampus 4, 44801 Bochum, Germany; 3 Medical Proteome Analysis, Center for Protein Diagnostics (PRODI), Ruhr-University Bochum, Gesundheitscampus 4, 44801 Bochum, Germany; 4 Institute of Physical und Theoretical Chemistry, Goethe-Universität Frankfurt am Main, Max-von-Laue-Str. 7, 60438 Frankfurt, Germany

**Keywords:** Chaperones, Kinases, Proteins, Mass spectrometry

## Abstract

Protein kinases are important regulators in cellular signal transduction. As one major type of Hsp90 client, protein kinases rely on the ATP-dependent molecular chaperone Hsp90, which maintains their structure and supports their activation. Depending on client type, Hsp90 interacts with different cofactors. Here we report that besides the kinase-specific cofactor Cdc37 large PPIases of the Fkbp-type strongly bind to kinase•Hsp90•Cdc37 complexes. We evaluate the nucleotide regulation of these assemblies and identify prominent interaction sites in this quaternary complex. The synergistic interaction between the participating proteins and the conserved nature of the interaction suggests functions of the large PPIases Fkbp51/Fkbp52 and their nematode homolog FKB-6 as contributing factors to the kinase cycle of the Hsp90 machinery.

## Introduction

The molecular chaperone Hsp90 is essential for cellular growth and via its client-base involved in cell metabolism, signal transduction, cell proliferation and apoptosis. The major Hsp90 clients are steroid hormone receptors and protein kinases. Both classes require association with Hsp90 and its cochaperones during their maturation and cellular function. A set of cochaperones, such as Hsp70 and Hsp40 contributes to their function. For each client class specific cofactors have been identified, like, p23, Hop and large PPIases for steroid hormone receptors and Cdc37 for kinases. Interactions between Cdc37 and Hsp90 have been reported both on the genetic^1,2^ and physical^3^ level and furthermore, have recently been confirmed in atomic detail^4^. Other cofactors of Hsp90, e.g., Aha1^5^ are also reported to influence the activity and complex formation with kinase clients, such as B-Raf. Whether more cofactors are involved in the kinase processing cycle of Hsp90 remains elusive.

ATP binding and hydrolysis is a central step in the Hsp90 cycle. This reaction induces dramatic conformational changes in the Hsp90 dimer and thereby influences the affinity of Hsp90 to its cochaperones^6^, which regulate the conformational cycle of Hsp90. The conformational changes of Hsp90 are mainly coupled to an N-terminal dimerization, which can be fully blocked by the cofactor Hop/Sti1^7^ and the Hsp90 inhibitory compounds geldanamycin and radicicol. Aha1 on the other hand accelerates the structural rearrangements and facilitates the dimerization. The cofactor p23 instead can decelerate the ATPase cycle of Hsp90^8^, by stabilizing the dimerized conformation. Also the interaction between Hsp90 and Cdc37 is modulated by ATP, as the presence of ATP reduces the affinity between Hsp90 and Cdc37^9^.

Protein kinases participate in complicated life cycles that include Hsp90-dependent regulation steps during their maturation and degradation. More than 40% of the known human kinases have been identified as client proteins of Hsp90 in mammals^10^. To date, kinase-associated chaperone complexes containing Hsp90 and its cofactor Cdc37, have been reported for many protein kinases, including B-Raf, v-Src and Akt^11–13^. Even though the importance of the Hsp90 chaperon system is well established, little is known about how the chaperone cooperates with its cofactors during the formation of these complexes.

Here, we use purified components to assemble Hsp90-complexes from nematode and mammalian origin with the solubilized kinase domain of B-Raf (sB-Raf). We analyze, which cofactors of Hsp90 additionally support the formation of sB-Raf•Cdc37•Hsp90 complexes, how nucleotide binding affects these complexes and how these complexes are organized in structural terms. Employing chemical crosslinking and LC–MS/MS, we reveal direct interaction sites among the chaperone proteins and sB-Raf helping to map the client binding sites in the chaperone and the contribution of the cofactors.

## Materials and methods

### Cloning, protein expression and purification

Human sB-Raf and other proteins were expressed from a pET expression vector with an N-terminal His_6_-tag in the *E. coli* strain BL21 (DE3) cod + . Cultures were incubated at 37 °C, induced with 1 mM IPTG at an OD_600_ of 0.6 and harvested 16 h post induction. For sB-Raf purification, cells were lysed in 25 mM Tris/HCl, pH 8, 1 mM DTT and 10% v/v glycerol. The lysate was clarified by centrifugation at 20,000 rpm for 45 min. sB-Raf was purified initially on a Resource S column by elution in 25 mM Tris/HCl, pH 8.0, 250 mM NaCl, 1 mM DTT, 10% v/v glycerol and further purified by size-exclusion chromatography on a Superdex 75 column in 25 mM Tris/HCl, pH 8, 1 mM DTT and 10% v/v glycerol. CDC-37, HSP-90, STI-1, FKB-6, PPH-5 and the components of the human Hsp90-system were captured on a HisTrap 5 mL FF column and then purified by gel filtration chromatography in 40 mM HEPES/KOH, pH 7.5, 20 mM KCl.

For deletion mutants, the structure of the FK506-binding motifs was estimated based on the published structure of Fkbp51^14^ which has a 42% sequence identity to FKB-6. Expression plasmids for ΔFK1 FKB-6 and ΔFK2 FKB-6 were generated using the Q5 mutagenesis kit with primers designed by NEBaseChanger (https://nebasechanger.neb.com/). Both FKB-6 variants were captured on a HisTrap column in 40 mM HEPES/KOH, pH 7.5, 1 mM EDTA, 1 mM DTT and eluted in imidazole containing buffer. Purification was achieved by ion exchange chromatography on a Resource Q column in 40 mM HEPES/KOH, pH 7.5, 1 mM EDTA, 1 mM DTT and elution from the Resource Q column in a linear gradient up to buffer containing 500 mM NaCl. Polishing of the FKB-6 variants was performed on a Superdex 75 size-exclusion column in 40 mM HEPES/KOH, pH 7.5, 1 mM EDTA, 1 mM DTT.

### Fluorescence labeling of sB-Raf variants

Cysteine residues of human Cdc37 and nematode CDC-37, as well as sB-Raf were labeled using ATTO 488 maleimide (ATTO-Tec, Germany). A twofold molar excess of the label was added to 0.5 mg protein in a buffer of 40 mM HEPES/KOH, pH 7.5, 20 mM KCl and 10% (v/v) glycerol. After an incubation time of 3 h at 20 °C, the reaction was stopped by adding 20 mM DTT and the free label was separated from the labeled protein by dialysis against the same buffer. The degree of labeling and the concentration of the protein were determined by UV/VIS spectroscopy using the following equations:

$$ \begin{gathered} {\text{A}}_{{{\text{Protein}}}} = {\text{ A}}_{{{28}0}} - {\text{A}}_{{{5}00}} \cdot \, \left( {{\text{CF}}_{{{28}0}} } \right) \hfill \\ {\text{DOL }} = {\text{A}}_{{{5}00}} \cdot {\text{MW}}/\left[ {{\text{protein}}} \right] \cdot \varepsilon_{{{\text{dye}}}} \hfill \\ \end{gathered} $$

where CF_280_ = 0.09 and ɛ_dye_ = 90,000 M^−1^ cm^−1^ according to the manufacturer. The degree of labeling for sB-Raf was found to be 1.12.

### Analytical ultracentrifugation

Sedimentation velocity experiments were performed on a Beckman ProteomeLab XLA analytical ultracentrifuge (Beckman Coulter, Brea) equipped with a fluorescence detection system (Aviv Biomedical, Lakewood, NY) and a Ti-50 rotor (Beckman Coulter, Brea) at 20 °C and at 42,000 rpm. 300 nM of labeled protein was analyzed in the absence and presence of 0.5 to 1 μM of its unlabeled putative binding partners or nucleotides at indicated concentrations. Measurements were performed in the storage buffer (40 mM HEPES/KOH, pH 7.5, 20 mM KCl, 1 mM DTT, and 5 mM MgCl_2_). dF/dt-plots from one rotor containing 14 samples were calculated by using the optimal time range of the experiment, subtracting the respective scans from each other and averaging over several such differentials with the in-house script diffUZ. The obtained averages were normalized against the plateau value of the fluorescence as described^15^. Plots were then fit to bi-Gaussian functions to get good estimates of the sedimentation coefficient s_20,w_ and peak amplitudes. All graphs were in Origin 8.6 (OriginLab).

### Crosslinking and purification of sB-Raf-containing complexes by SEC-HPLC

Chemical crosslinking of the complex proteins was performed with the isotope-labeled BS^3^-crosslinker H_12_/D_12_-BS^3^, containing hydrogen-containing and deuterium-containing BS^3^ at a ratio of 1:1 (Creative Molecules Inc.). 10 µM of sB-Raf, CDC-37, FKB-6 and HSP-90 were pre-incubated in crosslinking buffer (40 mM HEPES/KOH, pH 7.5, 50 mM KCl) for 5 min. The crosslinking reaction was initiated by adding 500 µM H_12_/D_12_-BS^3^. After 30 min of incubation at 25 °C, the reactions were stopped. To this end, 5 × SDS-PAGE sample buffer (250 mM Tris/HCl, pH 6.8, 10% SDS, 30% (v/v) Glycerol, 10 mM DTT, 0.05% (w/v) Bromophenol Blue) was added directly to the crosslinking reaction, if separation of complex species by SDS-PAGE was to be used. Images have not been cropped to remove lanes. For full-length analysis by mass spectrometry, instead, a Tris-buffer containing the reaction was stopped by addition of 1 M Tris/HCl, pH 8.0.

### LILBID-MS (laser induced liquid bead ion desorption mass spectrometry)

For stoichiometry evaluation the crosslinked sample and non-crosslinked mixtures of proteins were analyzed by LILBID-MS. A more detailed description of LILBID is given by Vitt et al*.*^16^ and Peetz et al*.*^17^. Briefly described, a piezo-driven droplet generator (MD-K-130 from Microdrop Technologies GmbH, Norderstedt, Germany) was used to produce droplets of 30 µm diameter with a frequency of 10 Hz at a pressure of 100 mbar.. Samples were loaded directly into the droplet generator and the generated droplets were subsequently transferred to high vacuum and irradiated by a mid-IR laser directly in the ion source. The laser employed was a Nd:YAG laser operating at 10 Hz, the wavelength being tuned by a LiNbO_3_ optical parametric oscillator to 2.94 µm ± 5 nm, the absorbing wavelength of the symmetric and asymmetric O–H stretching vibration of water. The pulse length was 6 ns with a maximum energy of 23 mJ. The laser power was measured by an optical power meter (PM100D, Thorlabs, Munich, Germany).

Droplet irradiation leads to an explosive expansion of the droplet containing the sample and solvated ions are released and analyzed in an in-house built time-of-flight setup operating at 10^–6^ mbar. The ion source is based on a Wiley-McLaren type design. The ions are accelerated into the grounded flight tube and guided towards the detector via a reflectron. The detector setup is based on a Daly-type detector optimized for the detection of high m/z ions. The voltage of the first (repeller) and second plate was set to -4 kV in the ion source. The third plate was grounded. The repeller was pulsed to – 6.6 kV for 370 µs after droplet irradiation. The pulse was applied between 2 and 20 µs after the droplet irradiation (delayed extraction time). The einzel lenses were set to − 3.0 kV. The reflectron was set to − 7.2 kV. Post-acceleration was set to + 17 kV at the MCP impact surface. Spectra processing was done by using the software *Mass*i*gn*^18^ based on *LabVIEW*.

All proteins were used in a 25 mM Tris, 20 mM KCl, pH 7.5 buffer except for sB-Raf, which was in 25 mM Tris, 250 mM NaCl, 1 mM DTT and 10% glycerol. The crosslinked sample was measured directly from the storage buffer, while the purified components were mixed with the ratio sB-Raf:CDC-37:FKB-6:HSP-90 = 1:1:1:1 or as indicated in the figure legend.

### Mass spectrometry

For identification of the crosslinked peptides, the crosslinking reaction was applied to SDS-PAGE. Relevant complex bands were then excised from the PA-gel and prepared for mass spectrometry. To this end, gel pieces containing the crosslinked protein bands were washed and destained three times by alternating 10-min treatments with 10 mM ammonium hydrogen carbonate (pH 8.3, buffer A) and buffer B (buffer A: 100% acetonitrile at a ratio of 50:50 (v/v)). After the destaining steps, samples were treated with 50 μL 10 mM DTT for 1 h at 56 °C and with 50 μL 50 mM IAA for 45 min at room temperature. Lastly, gel pieces were dried by vacuum centrifugation. The digestion to peptides was initiated by adding 8 μL of trypsin solution (0.015 µg/µl Serva, Heidelberg, Germany) and incubation was performed overnight at 37 °C. Peptides were eluted by incubating the gel pieces two times for 15 min with 30 μL of a 1:1 solution containing 100% acetonitrile and 0.1% (v/v) TFA in an ice-cooled ultra-sonication bath. Pooled supernatants were dried in a vacuum concentrator and resuspended in 20 μL 0.1% (v/v) TFA. The peptide concentration was determined by amino acid analysis (AAA) as described by Plum et al.^19^. Samples from the crosslinked complex of sB-Raf, CDC-37, HSP-90 dimer and FKB-6 were analyzed either on a Thermo Q Exactive Plus (dataset A) or Thermo Q Exactive HF (dataset B).

For dataset A, 5 µL of the samples were analyzed in a QExactive Plus mass spectrometer (ThermoFisher) coupled to an Ultimate 3000 nano HPLC system (Dionex). Samples were loaded on an Thermo Scientific Acclaim PepMap 100 C18 trap column (75 µm ID × 2 cm) and washed with 0.1% TFA. The subsequent separation was carried out on an Thermo Scientific Acclaim PepMap RSLC C18 column (75 µm ID × 50 cm) with a flow of 300 nL/min and buffer A: 0.1% formic acid in water and B: 0.1% formic acid in acetonitrile. Analysis started with washing in 5% B for 7 min, followed by a gradient from 5 to 22% buffer B within 105 min, an increase to 32% B within 10 min and another increase to 90% B within 10 min. 90% B was held for 10 min, then decreased to 5% within 0.1 min and held at 5% for another 9.9 min. The QExactive Plus mass spectrometer was run in a TOP10 data-dependent mode. Full MS scans were collected in a scan range of 300–1500 m/z at a resolution of 140,000 and an AGC target of 3e6 with 80 ms maximum injection time. The most intense peaks were selected for MS2 measurement with a minimum AGC target of 1e3 and isotope exclusion and dynamic exclusion (exclusion duration: 60 s) enabled. Peaks with unassigned charge or a charge of + 1 were excluded. MS2 spectra were collected at a resolution of 17,500 aiming at an AGC target of 1e5 with a maximum injection time of 100 ms. Isolation was conducted in the quadrupole using a window of 1.6 m/z. Fragments were generated using higher-energy collisional dissociation (HCD, normalized collision energy: 27%) and finally detected in the orbitrap.

For dataset B, 200 ng digested sample were measured by nano LC–ESI–MS/MS on an Q Exactive HF (ThermoFisher) as described previously^20^. An UltiMate 3000 RSLC nano LC system was utilized for nano HPLC analysis using the following solvent conditions: (A) 0.1% TFA; (B) 84% ACN, 0.1% FA. Samples were initially loaded on a trap column with a flow rate of 30 μl/min with 0.1% TFA. After sample concentration and washing, the trap column was serially connected with an analytical C18 column, and the peptides were separated with a flow rate of 400 nl/min using a solvent gradient of 4% to 40% B for 95 min at 60 °C. The HPLC system was on-line connected to the nano-electrospray ionization source of the mass spectrometer. The mass spectrometer was operated in a data-dependent mode with the spray voltage set to 1600 V in positive mode and a capillary temperature of 275 °C. Full scan MS spectra (mass range 350–2000 *m/z*) were acquired in the Orbitrap analyzer at a mass resolution of 60,000. The twenty most intensive ions per spectrum were subsequently fragmented using collision-induced dissociation (35% normalized collision energy) and scanned in the linear ion trap. The *m/z* values triggering MS/MS were set on a dynamic exclusion list for 30 s. After each sample measurement, 1 h of column washing was performed for equilibration.

### Analysis of the MS results

The raw data obtained from MS measurements were initially analyzed with MaxQuant 1.5 (https://maxquant.org/maxquant/)^21^ to confirm the presence of all proteins in the excised bands. Further all unmodified peptides and complete peak lists were obtained from the MaxQuant generated tables using the sequences of the crosslinked proteins. Statistics on the unmodified peptides confirmed the data quality (Supplemental Fig. 1). The peak lists yielded intensity values and elution times for each peptide. Then the peak lists were imported into the in-house software xMASS, which searches through peak lists to identify potential crosslinked peptide pairs that deviate in mass by 12.076 Da and can be assigned to a potential crosslinked product from a simulated crosslinked peptide library. The threshold was set to 6 ppm, 2 miss-cleavages were allowed in each crosslinked peptide. The hit list obtained was then filtered based on the type of crosslink and the peak intensities in parent scans. All crosslinks that originated from intermolecular peptide pairs and showed similar intensities for both isotope versions were further analyzed as to whether they matched the corresponding fragmentation spectra. In several cases, MS2 spectra originating from both, H_12_-BS^3^ and D_12_-BS^3^ crosslinking products, could be found, further confirming the identification.

**Figure 1:**
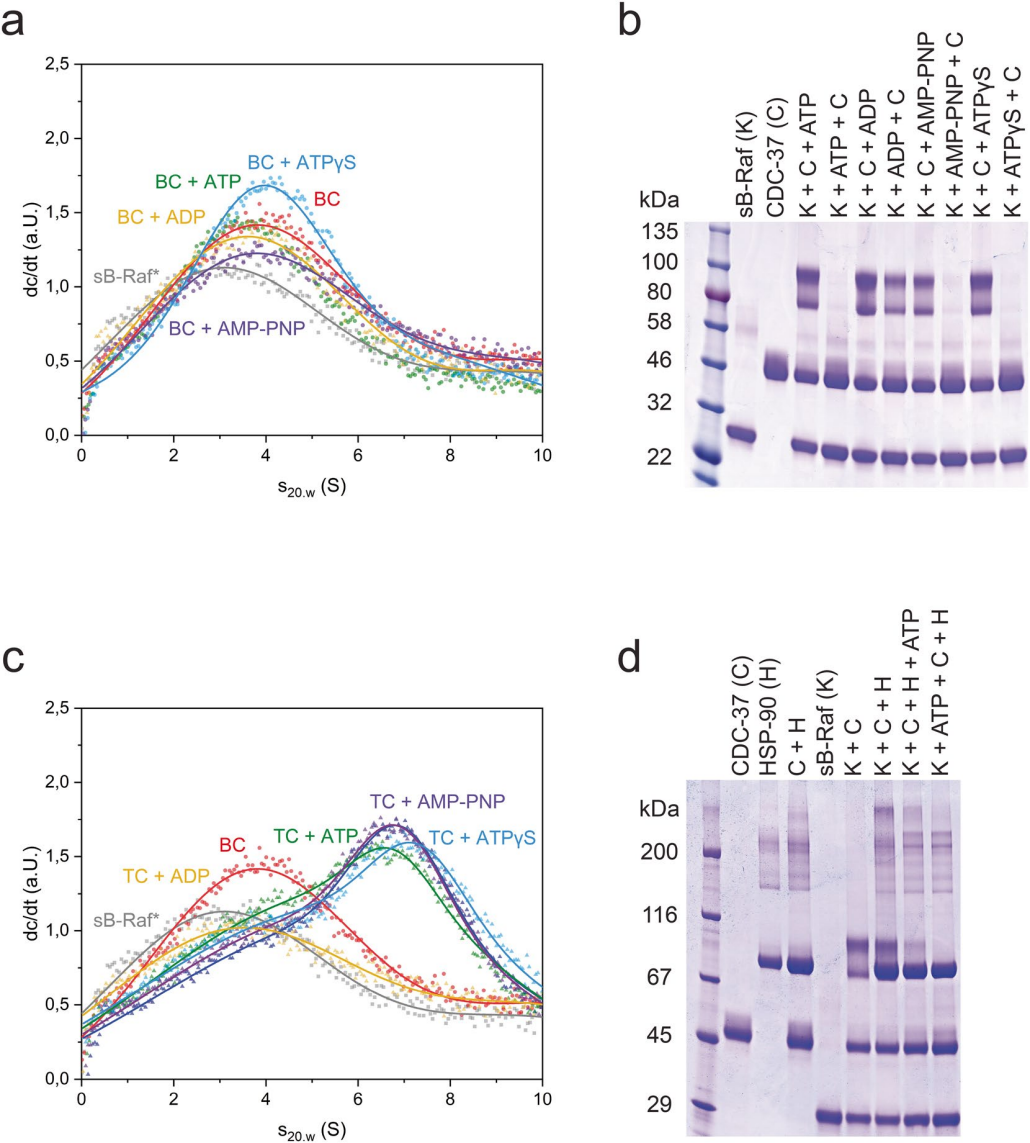
Influence of nucleotides on kinase complex formation. **(a)** Formation of sB-Raf•CDC-37 binary complex (BC) and influence of different nucleotides. **(b)** Influence of nucleotides on the binary complex formation when added in a different order. **(c)** Formation of sB-Raf•CDC-37•HSP-90 ternary complex (TC) and influence of different nucleotides. **(d)** Antagonism between ATP and chaperons in ternary complex formation.

For comparison this analysis was also performed with the software pLink [http://pfind.ict.ac.cn]^22^. The analysis was done with the following settings: Peptide mass range: 400–6000 Da; Peptide length: 4–60 AA; precursor tolerance: ± 10 ppm; fragment tolerance: ± 10 ppm; filter tolerance: ± 5 ppm; FDR < 5% at PSM level. Reported crosslinked spectra were filtered to a mass error < 2 ppm and then compared to the xMASS outputs. The confirmed hits are listed in Supplemental Table 1. Sequence alignments of human and nematode proteins were performed in Benchling (https://benchling.com).

The visualization of all crosslinking results was performed on the webserver xiNET^23^.

### Homology modeling and model evaluation

Homolog modeling of the ternary complex containing the nematode proteins HSP-90, CDC-37 and sB-Raf was performed with Chimera coupled to MODELLER and was based on the PDB structure 5FWL. For refinement of the sB-Raf structure, the PDB file 5CSW was used. All obtained models were refined in Chimera with default settings. The model was validated by comparing the obtained crosslinks between the three proteins to predicted crosslinking distances within this structure calculated by Xwalk (http://www.xwalk.org/). Distances between crosslinked residues were measured in Pymol (https://pymol.org/2/). The spacer length of the crosslinking reagent was used to align the respective dimeric part of HSP-90 as well as the cofactors to the complex structure, assuming only one cofactor being present as determined by native mass spectrometry.

## Results

### Nucleotides antagonize against protein chaperons during complex formation

Nematode HSP-90 and CDC-37 form protein complexes with the stabilized kinase domain of sB-Raf and the complex has been found to be sensitive to the presence of ATP in previous studies^9^. To further understand the influence of nucleotides on the association of this important HSP-90•client complex, ATTO488-labeled sB-Raf (*sB-Raf) was generated and used to investigate the complex formation. *sB-Raf sediments as a monomeric protein at 2.8 S in sedimentation velocity AUC experiments (Fig. 1a). The addition of CDC-37 to the labeled kinase initiates the complex formation, which is observable by an increase of the sedimentation coefficient (s_20,w_) of *sB-Raf from 2.8 S to 4.3 S (Fig. 1a). This is in agreement with the heterodimeric complex observed before with fluorescently-labeled *CDC-37 and unlabeled sB-Raf^24^.

To investigate the influence of nucleotides on the regulation of this binary complex, analytical ultracentrifugation was applied in the presence of different nucleotides. This can yield information on different steps during ATP turnover by the kinase. The sedimentation coefficient of *sB-Raf, which is increased in the presence of CDC-37, is sharply reduced in the presence of ADP, but AMP-PNP and ATPγS do not influence the interaction within the complex. To confirm this observation also with unlabeled proteins, we performed chemical crosslinking with the crosslinker BS^3^ and investigated the size of the crosslinking products by SDS-PAGE (Fig. 1b). The crosslinked complex can be readily observed, while the two proteins individually do not form a similar-sized crosslinking product. We then tested nucleotide addition to see, whether the inhibitory influence of ATP, ADP and ATP-analogs can likewise be observed. Indeed, the crosslinking between CDC-37 and sB-Raf is disrupted in the presence of ATP, implying reduced affinity between the interaction partners. The complex between CDC-37 and sB-Raf likewise can be disrupted if ADP, AMP-PNP or ATPγS is added to sB-Raf kinase prior to the crosslinking reaction. Surprisingly, we find this behavior to depend on the order of addition: If nucleotides are added to sB-Raf before addition of CDC-37, the sharply reduced crosslinking efficiency is observed. If instead CDC-37 is added to the kinase first and nucleotides are added thereafter, the binary complex formation is not blocked. The reduced complex formation upon nucleotide addition corresponds to results described in a study by Polier et al., which finds that Cdc37 interaction is impacted by nucleotides in a similar fashion^25^. Our results suggest that the conversions modifying the binding behavior in the nucleotide-complexed kinase domain or the CDC-37-bound kinase domain are slow processes, as apparently a once formed complex between CDC-37 and sB-Raf is only slowly reduced to an extent that crosslinking is prevented.

It has been well-described that CDC-37 delivers the HSP-90 dependent kinase to the molecular chaperone^10^. We therefore tested, whether the influence of nucleotides is also observed, if *sB-Raf and CDC-37 are in a complex with HSP-90 (Fig. 1c,d and Supplemental Fig. 2). The formation of the full ternary protein complexes with an s_20.w_ of 6.5 S had been observed using fluorescently-labeled CDC-37 before^12^. This complex with labeled *sB-Raf was less sensitive to nucleotides compared to the binary complex of *sB-Raf and CDC-37 alone. While the addition of ADP leads to dissociation of the complex, the addition of ATP, AMP-PNP and ATPγS does not prevent the formation of the ternary complex and ATPγS even increases the sedimentation coefficient of the labeled *sB-Raf to 7.0 S (Fig. 1c). This increase suggests that under these conditions, the closing of the HSP-90 chaperone may be induced, and the dissociation is prevented. This highlights the complexity of interactions in this protein assembly, where both, kinase and HSP-90, can bind nucleotides. Interestingly, here likewise the order of nucleotide and CDC-37 addition was found to be important for complex assembly: If ATP is added to the kinase prior to the addition of CDC-37 and HSP-90, chaperone complexes are not assembled at all. If instead ATP is added after the formation of the chaperone complex, the protein complex appears not fully dissociated, but apparently reduced by the addition of ATP (Fig. 1d).

**Figure 2:**
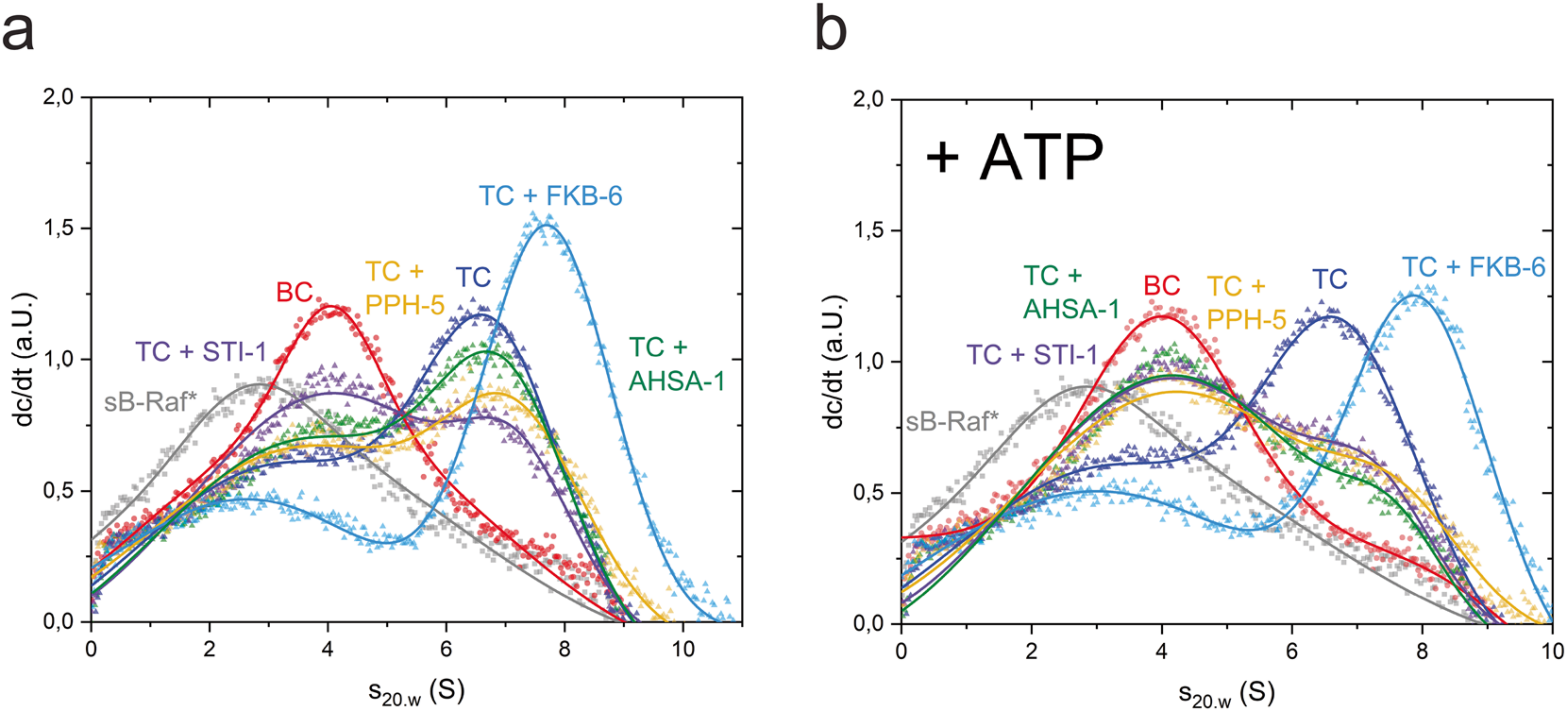
Influence of HSP-90 cochaperones on the sB-Raf•CDC-37•HSP-90 ternary complex. **(a)** Changes of sB-Raf•CDC-37•HSP-90 ternary complex (TC) induced by the presence of HSP-90 cochaperones. **(b)** Influences of ATP on complexes formed with HSP-90 cochaperones. BC stands for sB-Raf•CDC-37 binary complex.

### Participation of FKB-6 in the HSP-90•CDC-37•sB-Raf complex

HSP-90 requires cooperation and assistance from its cofactors to fulfill its function. We tested whether additional cofactors of HSP-90 can facilitate the formation of the kinase complex. To this end, the complex formation was investigated in the presence of the HSP-90 cofactors AHSA-1, STI-1, PPH-5 and FKB-6, all of which have been reported to interact with HSP-90 as cofactors in nematode and human systems^26–29^.

We added each of the cofactors individually to the preformed ternary complex and observed the induced changes in AUC-experiments. The addition of AHSA-1 reduced the amount of the sB-Raf•CDC-37•HSP-90 ternary complex and so did STI-1, but to a lower extent (Fig. 2a). in both cases no quaternary complex can be observed. PPH-5 also reduced the amount of CDC-37•HSP-90 bound to sB-Raf, but at the same time enlarged the sedimentation coefficient of the remaining complex, showing that PPH-5 can bind in addition to preformed assemblies of HSP-90, CDC-37 and the kinase domain. In contrast to the other cofactors, FKB-6 joined the sB-Raf•CDC-37•HSP-90 complex by forming a stable protein assembly at 7.8 S. The strong increase in sedimentation coefficient and the sharp decrease in sB-Raf•CDC-37 at 4.8 S shows that the binding of FKB-6 is strongly supportive to the interaction between sB-Raf and the HSP-90 system.

We next tested the influence of ATP on the formation of these quaternary complexes (Fig. 2b). For all HSP-90 cofactors with exception of FKB-6, addition of nucleotides leads to destabilization of the ternary or quaternary chaperone complex. This is evident from the reduction of the complex peaks at higher sedimentation coefficient. Only in the case of FKB-6, the ternary complex is not destabilized by the presence of ATP, but rather shifts towards a slightly larger sedimentation coefficient (s_20,w_ = 8.2 S), which might indicate the dimerization of HSP-90's N-terminal domains that is known to occur in response to ATP and ATP-analogs during client processing. Other nucleotides were tested with the quaternary complex as well (Supplemental Fig. 3). There likewise, after addition of AMP-PNP or ATPγS, protein complexes are observed at increased sedimentation coefficients, hinting at the ability of these nucleotides to induce the closing reaction of HSP-90. This suggests that the compaction usually observed for HSP-90 alone apparently is also possible in the presence of kinase client and the two HSP-90 cofactors FKB-6 and CDC-37.

**Figure 3:**
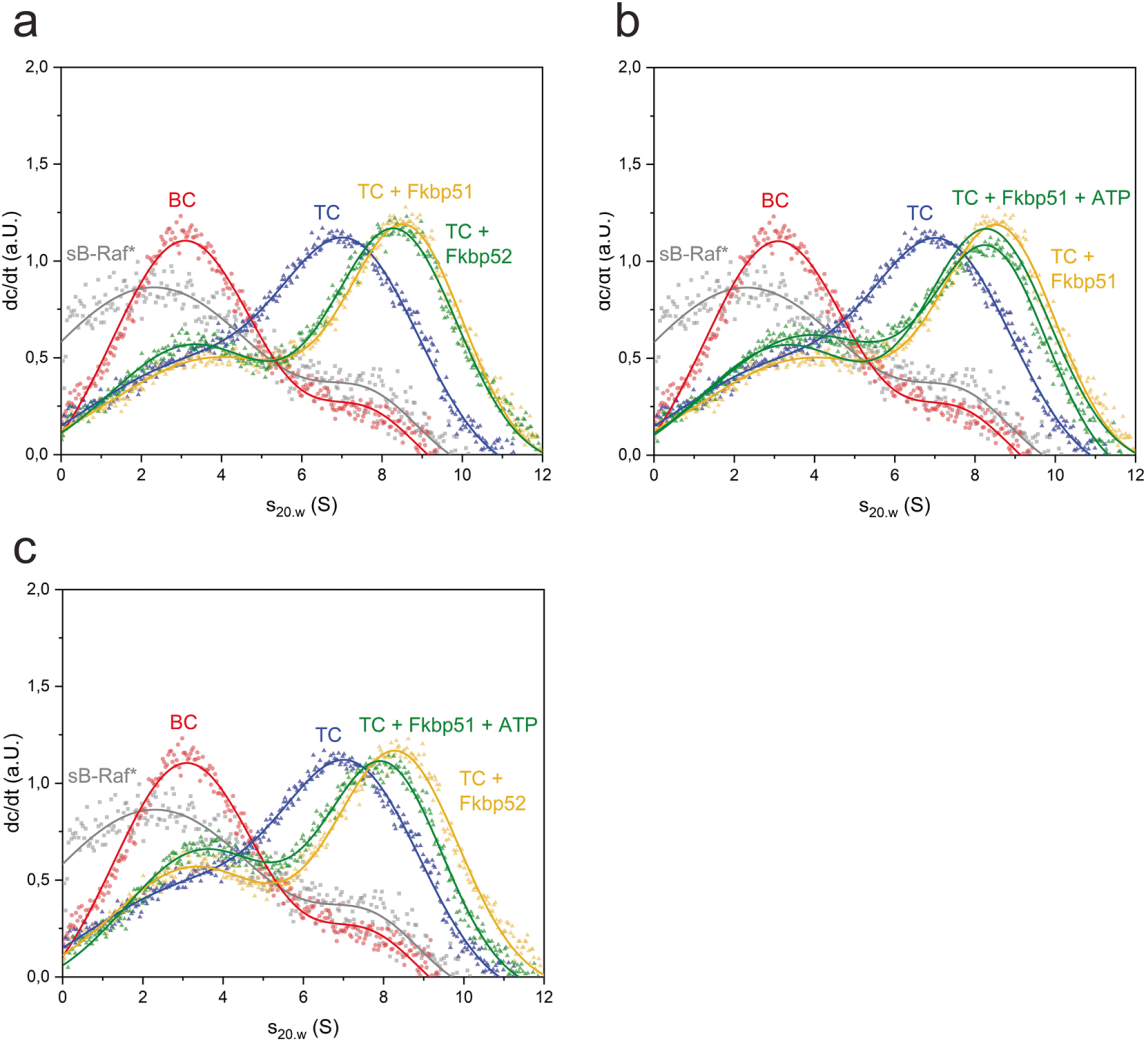
Formation of quaternary complex in the human system. **(a)** Formation of quaternary complex with human homolog chaperons Fkbp51 and Fkbp52. **(b)** Influence of nucleotides on the quaternary complex with Fkbp51. **(c)** Influence of nucleotides on the quaternary complex with Fkbp52. sB-Raf•Cdc37 binary complex is abbreviated with BC, whereas TC stands for the sB-Raf•Cdc37•Hsp90 ternary complex.

### Human Fkbp-like proteins integrate into Cdc37•sB-Raf•Hsp90 complexes

In the human Hsp90 system, the participation of the large PPIases homologous to FKB-6 has been studied extensively in the context of steroid receptor clients, but not for kinase-containing chaperone complexes. Therefore, we tested whether a similar interaction can be observed in the mammalian system. Contrary to the nematode chaperone system, which only contains FKB-6, the human system has two homologs. We therefore tested the purified human PPIases Fkbp51 and Fkbp52 and their interaction with Hsp90, sB-Raf and human Cdc37 in sedimentation velocity AUC experiments.

The ternary complex consisting of *sB-Raf, Hsp90β and human Cdc37 was formed in analogy to the nematode protein complex. Addition of the PPIases Fkbp51 and Fkbp52 demonstrates their ability to integrate into the complex. Both, Fkbp51 and Fkbp52, form a stable quaternary complex with *sB-Raf•Cdc37•Hsp90β, increasing the sedimentation coefficient of the complex to 9 S from 7.2 S for the ternary complex (Fig. 3a).

The influence of ATP was tested in the human system as well (Fig. 3b,c). Contrary to the results from the nematode system (see Fig. 2b), the addition of ATP weakens the quaternary complex and leads to a reduced s_20,w_ value of the labelled *sB-Raf in complex with the chaperone system. This may show, like in many studies before, the reduced ability of the human Hsp90 system to form the N-terminally dimerized state in response to nucleotide binding and that this trait also is maintained in the quaternary complex.

### Both FK506-binding domains of FKB-6 contribute to the complex stability

**Figure 4:**
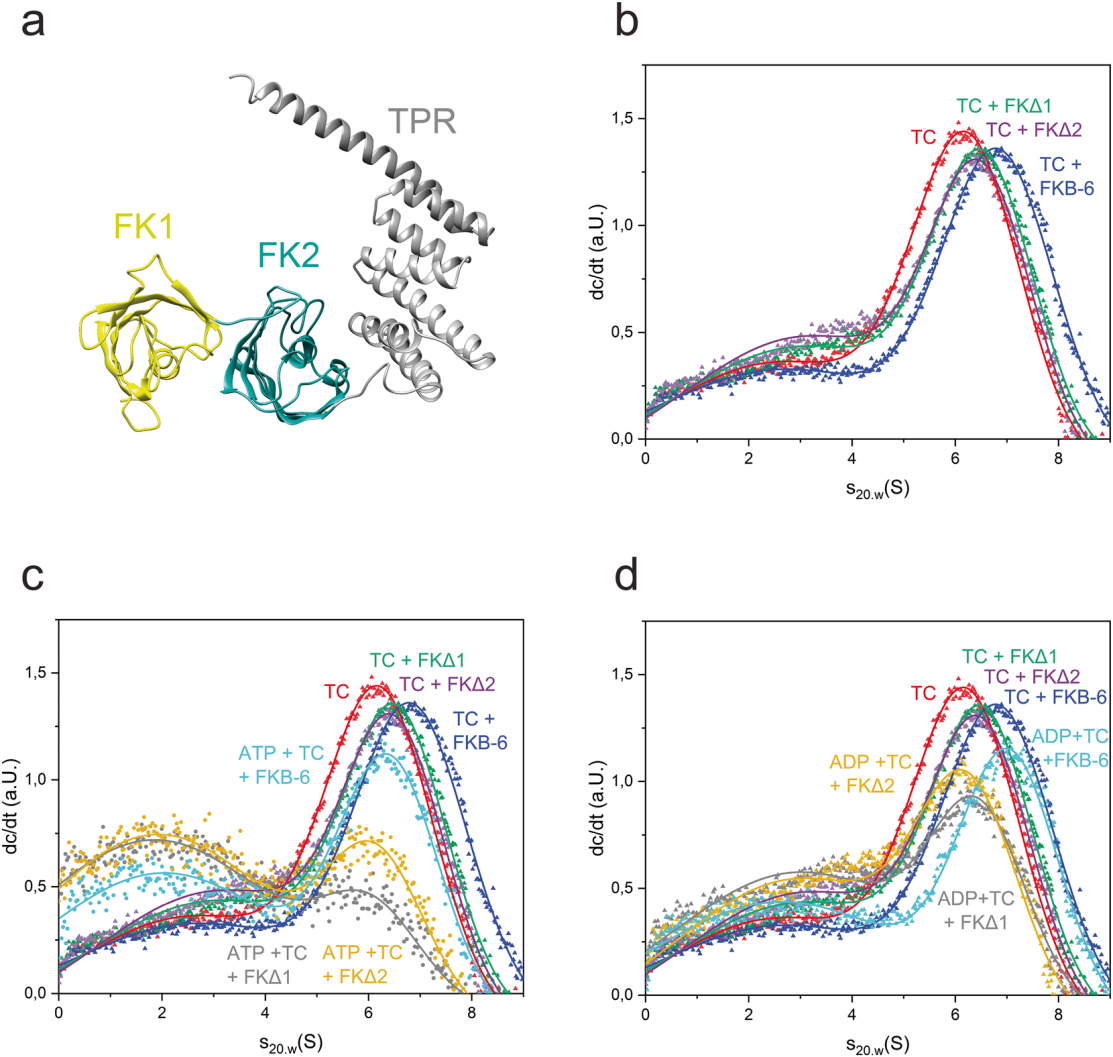
Role of Fkbp domains on complex formation. **(a)** Structure of FKB-6 human homolog Fkbp51 (PDB: 1KT1), made in Chimera 1.10.2. First Fkbp domain: yellow, second Fkbp domain: light blue, TPR domain: gray. **(b)** Comparison of the complex formed with FKB-6 and its mutants. Antagonisms between **(c)** ATP; **(d) **ADP and chaperons in ternary complex formation.

In both, *C. elegans* and human systems, FKB-6 or homolog PPIases are able to stabilize sB-Raf containing complexes. To obtain insight into the mechanism of this cooperative interaction, the contribution of individual FKB-6 domains was addressed. FKB-6 consists of two FK506-binding domains and a C-terminal TPR domain. We designed constructs of FKB-6 with deleted FK506-binding domains and investigated the performance of these variants in quaternary complex formation. Besides a deletion of the first FK506-binding domain (∆FK1-FKB-6), a deletion of the second FK506-binding domain (∆FK2-FKB-6) was performed by including a flexible (GGGGS)_3_ linker to bridge the deletion of the domain between FK1 and the C-terminal TPR-domain (Fig. 4a).

SV-AUC results with these deletion variants imply that both FKB-6 mutants are still able to bind to the ternary complex in a similar manner, with a slightly smaller s_20,w_ compared to the full length FKB-6 protein (Fig. 4b). As FKB-6 primarily interacts with HSP-90 via its TPR domain, the two FKBP-domains apparently contribute in the same manner. We then tested how the depleted constructs respond to the addition of different nucleotides. In the presence of ATP, the ability of both FKB-6 variants to maintain *sB-Raf in the chaperone complex is dramatically reduced compared to full-length FKB-6, as derived from the increased presence of free labeled *sB-Raf at a sedimentation coefficient of 2.5 S (Fig. 4c). Complex dissociation is similar for both deletion constructs and stronger for ATP than for ADP addition (Fig. 4d). In contrast to the deletion constructs, the full-length FKB-6, retains sB-Raf•CDC-37 in the chaperone complex and induces the closed state of HSP-90. Thus, both FK506-binding domains of FKB-6 contribute to the stability of the kinase-chaperone complex.

### Stoichiometry of protein components in the sB-Raf-chaperone complex

**Figure 5:**
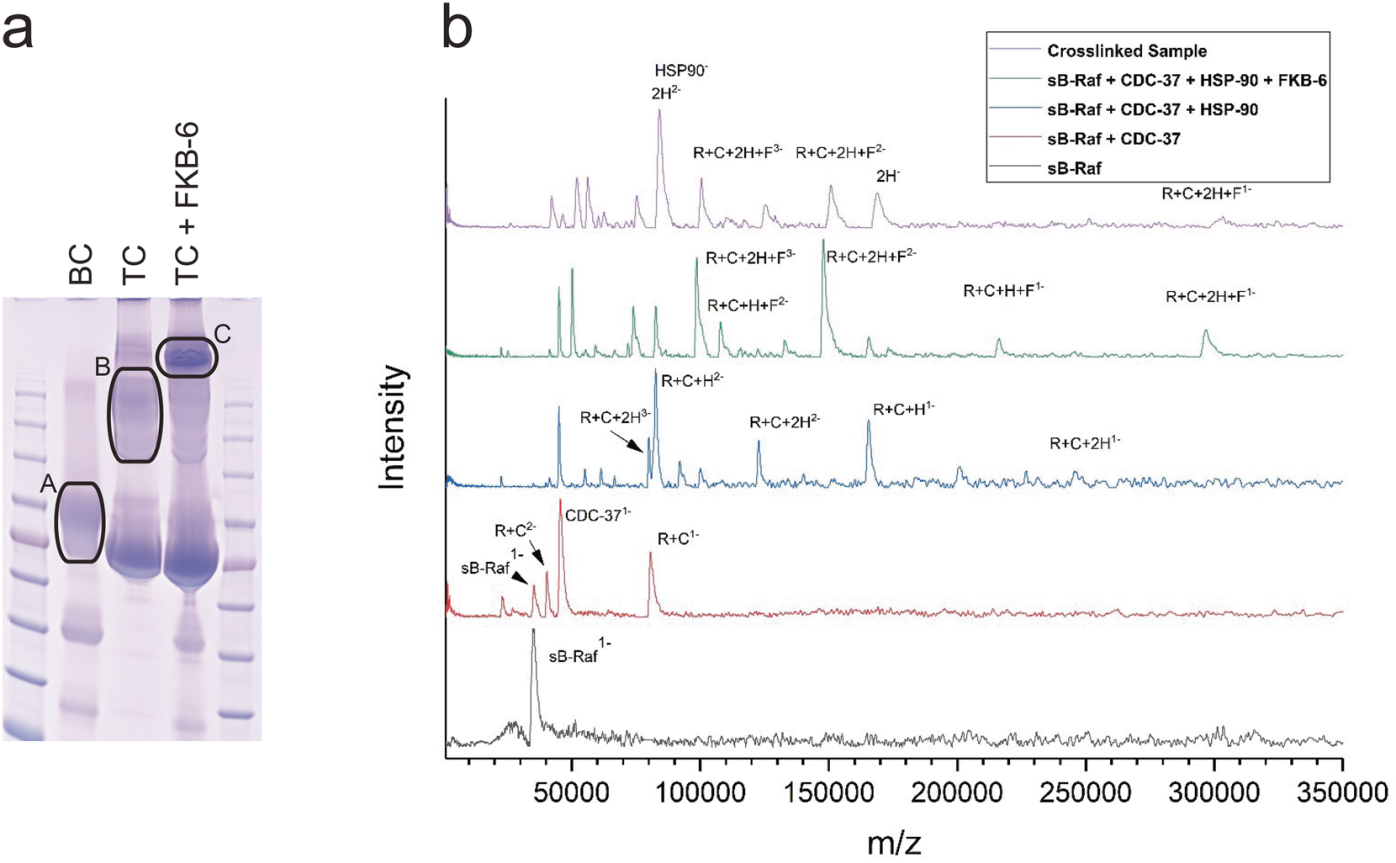
Stoichiometry of Raf-complex formation. **(a)** Impact of single components by crosslinking on the complex formation. BC = sB-Raf with CDC-37; TC = BC with the addition of HSP-90 dimer. The labeled gel area is used in later MS analysis. **(b)** Native MS of crosslinked sample from **(c)** vs single components, R = sB-Raf; C = CDC-37; D = HSP-90; F = FKB-6.

To gain more information on the stoichiometries within the quaternary protein complex, we investigated the complex by full-length and native mass spectrometry. The stoichiometry of Cdk4•Cdc37•Hsp90 had been previously determined by negative-staining electron microscopy and cryo-electron microscopy to be 1:1:2^30^. To investigate the quaternary complex including FKB-6, we compared native protein mixtures with the crosslinked protein samples under conditions that allow full-length and native mass spectrometry^17,31^. This is difficult to study by SV-AUC, as the dynamic within the protein complex and the incomplete saturation does not allow to get conclusive results, even if titration experiments are performed (Supplemental Fig. 4). In crosslinked samples (Fig. 5a, Lane C) several species can be observed (Fig. 5b, upper spectrum) and some of them can be assigned to defined chaperone complexes based on their m/z ratios and with the help mixtures of the native components (Fig. 5b, lower spectra). Based on the computed mass, the largest observed species corresponds to the complex sB-Raf•CDC-37•FKB-6•HSP-90 at a stoichiometry of 1:1:1:2. Upon exclusion of FKB-6 from the native protein mixtures, this species is not observable anymore and the complex of sB-Raf•CDC-37•HSP-90 is observed as dominant species based on the molecular mass. Also, the 1:1 complex of sB-Raf and CDC-37 is detectable in the respective mixture. The crosslinked and the assembled protein samples therefore suggest a preferential formation of protein complexes consisting of one client, one set of cofactors and the dimeric HSP-90 machinery.

### Structural organization of the sB-Raf•CDC-37•FKB-6•HSP-90 complex

Having obtained the preferential stoichiometry of the protein complex, we used the crosslinked quaternary complex to identify relevant interaction sites in the crosslinked protein complex. To this end we performed a crosslinking reaction of the full quaternary complex, the binary complex sB-Raf•CDC-37 complex and HSP-90 alone as reference (see Fig. 5b). We employed two different mass spectrometry setups and performed an exhaustive identification of crosslinking sites from the obtained datasets.

**Figure 6:**
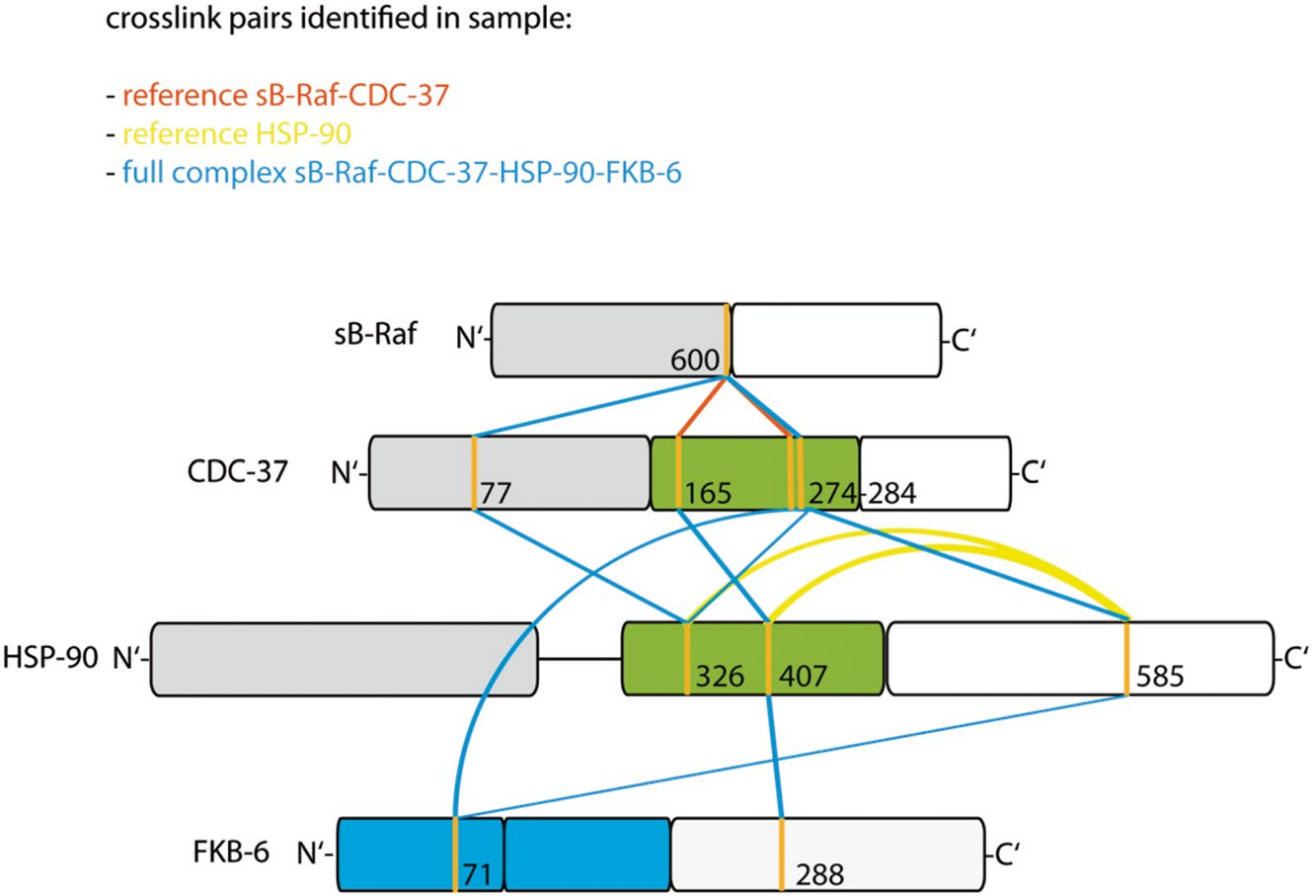
Structural information obtained from MS analysis. Schematic diagram of key crosslinked pairs in the complex. Red lines indicate crosslink in the sample sB-Raf•CDC-37; Yellow lines indicate pairs identified in the HSP-90 sample; Blue lines indicate pairs from the complex sample. N-terminal domains are colored in gray; C-terminal domains in white; Middle domains in green and FK-domains in blue.

**Figure 7:**
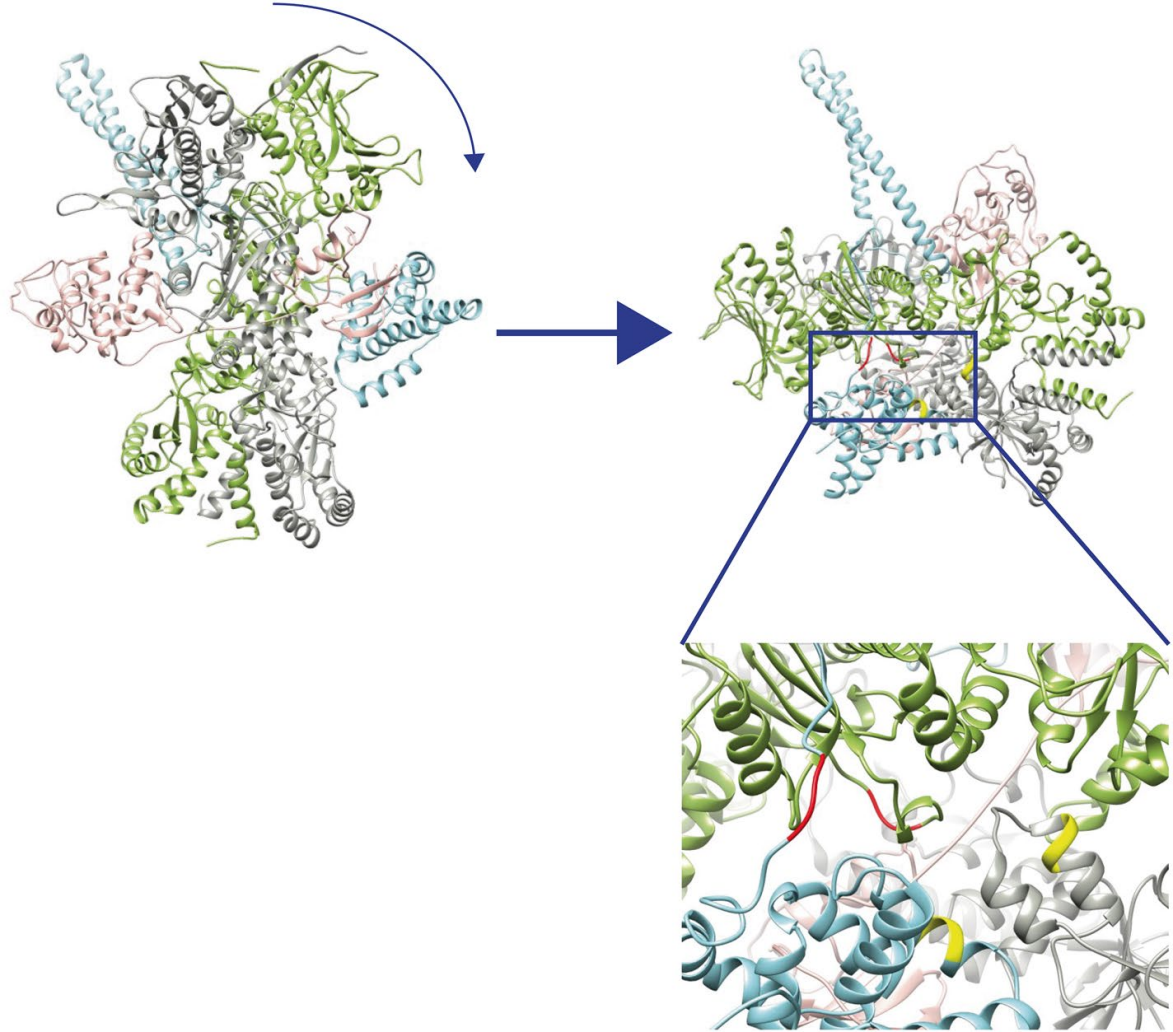
Homology model of kinase chaperon complex. The model is generated in Chimera 1.10.2 based on the PDB structure 5FWL. Kinase domains are indicated as light pink; CDC-37 as light blue; HSP-90 monomer A is indicated as dim gray; HSP-90 monomer B as light green; Conserved CDC-37•HSP-90 crosslinked residues are highlighted in red (Pair D) and yellow (Pair E).

For both datasets, two independent analysis programs, in-house xMASS and pLink, were employed and delivered comparable results. Mapping of the location of all identified crosslinked peptide pairs indicates that the two mass spectrometry approaches revealed similar crosslinks in the two experiments. We detected unmodified peptides and intramolecular and intermolecular crosslinked peptide pairs. The unmodified peptides clearly showed the presence of FKB-6, CDC-37, sB-Raf and HSP-90 in the crosslinked complex and revealed the high quality of the mass spectrometry data at 3 ppm. While the intramolecular crosslinks were used to confirm the homology modelled structure (Fig. 7), the intermolecular crosslinks between FKB-6, CDC-37, HSP-90 and the kinase domain were used to obtain the relative arrangement of these proteins in the complex (Fig. 6).

The most prominent intermolecular crosslinked peptides identified in the three samples are listed in Supplemental Table 1. An overview of intermolecular crosslink sites (Supplement Fig. 1) shows that crosslinks between all four proteins could be identified by this approach.

In the quaternary complex we first identified crosslinks between CDC-37, HSP-90 and sB-Raf, as for this complex already a structural model exists based on an electron microscopic structure^4^ and a cryo-electronic structure^30^. Intermolecular crosslinks between these proteins link the middle domain of CDC-37 at position 274 to the lobe-domain of sB-Raf (IGDFGLATVKSR, AA600), implying that these regions are in contact in the HSP-90-containing structure. Another contact of this kinase position could be formed with the N-terminal domain of CDC-37 at peptide MAEKKMEQEK (AA77) and likewise in the binary complex with peptide KPQAPK (AA165). Also in the binary complex sB-Raf•CDC-37, two intermolecular crosslinked pairs between sB-Raf (IGDFGLATVKSR, AA600) and the CDC-37 peptides KPQAPK (AA165) and QFFKK (AA273) were identified. As both crosslinks between CDC-37 and kinase domain locate in the M-domain of CDC-37 (Supplemental Table 1a), it can be assumed that in the binary complex and also in the quaternary complex the middle domain of CDC-37 approaches the interface between the N-and C lobe of the kinase.

30 of the 37 intermolecular crosslinks are linking CDC-37 and HSP-90 (Supplemental Table 1c). The interaction between CDC-37 and HSP-90 is characterized by interactions between the M-domains of both proteins at CDC-37 (KFEAAEPVYMK, AA274) and HSP-90 (APFDLFENKK, AA326). Further contact sites in the middle domains are the CDC-37 peptide KPQAPK (AA165) crosslinked to the HSP-90 peptide KFYEQFGK (AA407). Also prominent is a crosslink between KFEAAEPVYMKHYQDEVK (CDC-37, AA284) and IMKAQALR (HSP-90, AA585). As all these peptides are positioned in the cryo-electron-microscopic structure we modelled the structure for the proteins utilized by us. We then tested, to what extent the identified crosslinks are in proximity in this structure. All CDC-37•HSP-90 crosslinked residues in the ternary complex are calculated to be in the range of 8–17 Å away from each other, which corresponds nicely to an ideal distance for the crosslinking reactions. Nevertheless, it is challenging to assign the Hsp90 chain to the crosslinking site (chain A is shown in dim gray and chain B in light green in Fig. 7). With the help of the homology model, it can be assumed that the residue 407 of HSP-90 chain B is forming the crosslinked pair, as the residue 407 of HSP-90 chain A is spatially separated from the reaction partner. For the same reason, it is to be assumed that the residue 585 of HSP-90 chain A is crosslinked to residue 284 of CDC-37. Unfortunately, the kinase structure within PDB 5FWL is not well resolved, leaving large parts of the sequence unaccounted for. Some crosslinked pairs involving kinase residues are found to be separated from each other with a distance over 30 Å, implying that in some cases structural rearrangements may have to be considered.

Positioning the TPR-cofactor FKB-6 close to CDC-37 in this complex is supported by the identification of the crosslink between RAEEVLEYEKSTDPEK (FKB-6, AA288) and KPQAPK (CDC-37, AA165), which positions the FK2 domain of FKB-6 into the vicinity of the middle domain of CDC-37. Further crosslinks of FKB-6 are linking the FK1-domain to CDC-37 with FEAAEPVYMKHYQDEVK (CDC-37, AA284) and GNVIKGWDLGVATMTK (FKB-6, AA71). In addition, two further crosslinked peptides of FKB-6 are identified. FKB-6 (RAEEVLEYEKSTDPEK, AA288) is crosslinked to HSP-90 at peptide KFYEQFGK (AA407). Another interaction is observed between the FK1 domain of FKB-6 (GNVIKGWDLGVATMTK, AA71) and CDC-37 (FEAAEPVYMKHYQDEVK, AA284). These crosslinks imply that FKB-6 shares interaction sites with both proteins in the quaternary complex, which may contribute to the cooperativity observed in context with this interaction.

Regarding the relative position, the structure of the *C. elegans* complex is calculated based on homology models of the human homolog complex 5FWL. The models are built based on real contact sites. The model assumes that the arrangement of side chains or protein backbone locations are unchanged. Under these assumptions the homology model offers information on areas which are closely localized to each other. The comparison of the models indicates that main interaction sites between HSP-90 and CDC-37 are conserved. Kinase-chaperone interaction sites in the human homolog structure might differ from those in the *C. elegans* model, as sB-Raf shares only 25% of sequence identity with Cdk4 in the kinase domain sequence, which implies potential differences in certain subdomains not respected during homology modeling. Despite this, the quality of the homology modelled structure model can be validated by crosslinked peptides between HSP-90 and CDC-37 and the distances of the crosslinked positions are in agreement with the 30 Å maximal length bridged by the crosslinking compound.

## Discussion

### Nucleotides and CDC-37 antagonize against each other in complex with sB-Raf kinase

The molecular chaperone HSP-90 interacts with ATP and its cochaperones. In the context of the kinase cycle, the importance of these interactions is far less clear than in the context of other HSP-90 clients like steroid receptors, where ATP-binding and hydrolysis by HSP-90 contribute to the generation of the steroid binding competent receptor. The data presented here give an indication of the importance of nucleotides and cochaperones for HSP-90 complexes with protein kinases.

Based on our previous results^9^, nucleotides and chaperones interact with the kinase in an antagonizing way. When sB-Raf is in complex with ATP or ATP homologs, it is unable to bind to CDC-37. ADP has less influence on the structure of the kinase domain, as it does not to affect the interaction with CDC-37 to the same extent. Instead, binding of ATP activates the kinase by initiating conformational rearrangements to orient the catalytic region in the kinase domain. This is well described at least for the kinase PKA and likely similar in other kinases^32^. In contrast, ADP does not contribute to the assembly of the catalytic region, hence maintaining the kinase in an apo-like state. The reduced affinity thus may result from conformational rearrangements of the kinase domain in response to ATP. Once the rearrangement has been performed, the kinase is activated and able to carry out its function, at which state it may not require the further assistance of the chaperone system. This may be an indication of how chaperons selectively bind the relevant clients.

Interestingly we identify several crosslinking products linking the lobe domain of the kinase to the CDC-37 middle domain. With these lobes being affected by ATP-binding it is well possible that the rearrangements in the kinase domain after nucleotide binding hide the interaction surfaces utilized by CDC-37 for complex formation.

However, once HSP-90 is bound to the kinase client, the nucleotide-induced effects in the kinase are reduced, potentially via blocking central residues in the catalytic spine or further unfolding the kinase domain as described for the human system by Verba et al*.*^30^, where the N-lobe and C-lobe of the Cdk4 kinase domain are pulled apart, when in the complex with Hsp90.

Since both, Hsp90 and kinase, are able to bind and hydrolyze ATP, the nucleotide-induced regulation within the complex is opening a complex question. Therefore, it will now be interesting to see, how the assays developed in this study may facilitate the analysis of protein variants with altered mechanistic behaviour. Based on previous studies it is clear that further steps during the activation cycle have to be considered in order to obtain the full picture of the interaction. These steps also include phosphorylation events on the cofactors and the chaperone by the kinase and further regulatory steps that are influenced by phosphorylation or other post-transcriptional modifications on each of the contributing factors. It will be interesting to see weather PTMs impact both formation and function of the sB-Raf•CDC-37•FKB-6•HSP-90 complex, since all of the proteins in this complex are potentially regulated by PTMs on their own as recently shown in the human Hsp90 system. This additional level of regulation, termed the "chaperone code", may influence every aspect of Hsp90 interactions and functions^33,34^.

### FKB-6 stabilizes the kinase•CDC-37•HSP-90 complex against antagonistic interaction with ATP.

**Figure 8:**
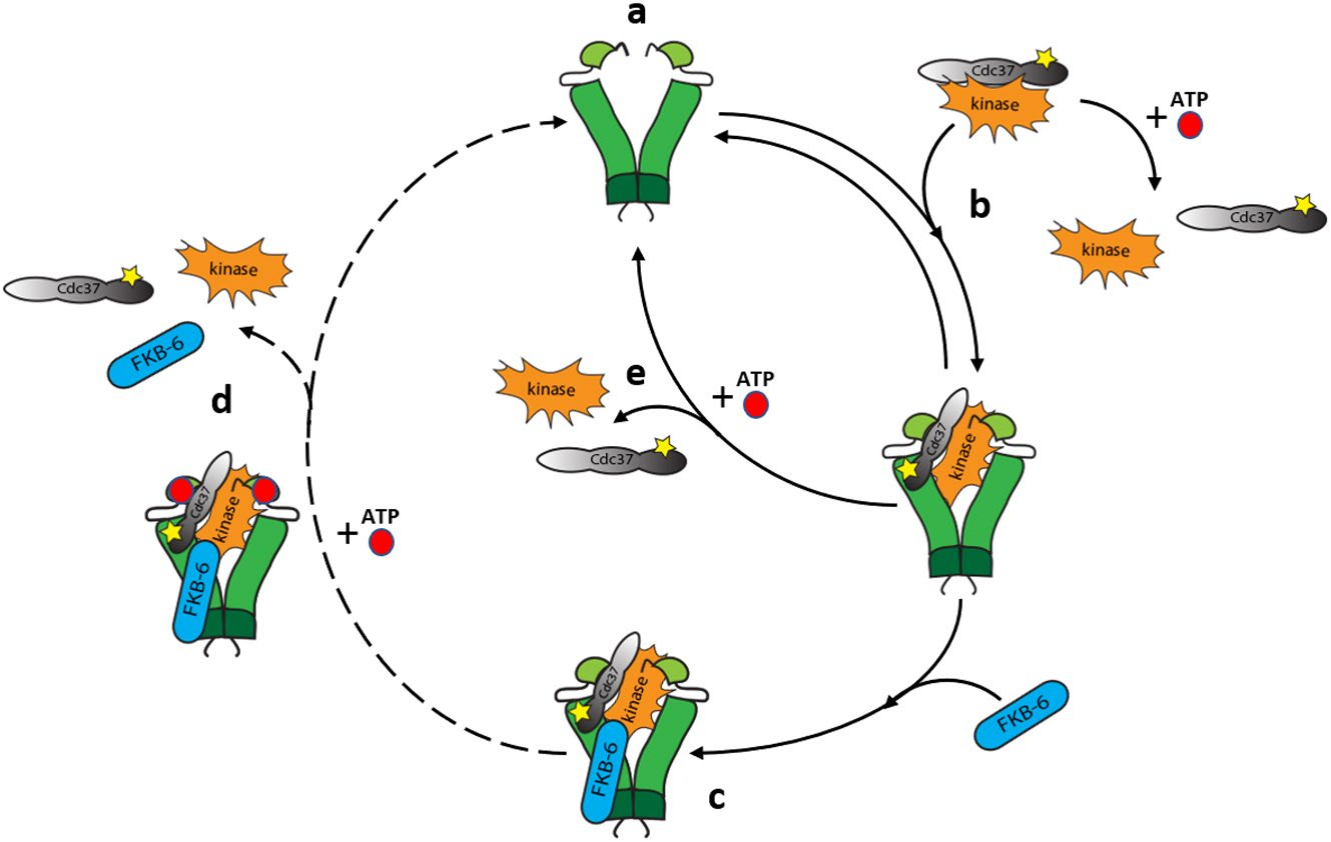
Graphical presentation of FKB-6 involved in the kinase cycle of HSP-90. HSP-90: Green; CDC-37: Grey; Kinase: Orange; FKB-6: Blue; ATP: Red; Dashed line: very slow event with potentially further steps, including phosphorylation of one or more of the components.

The equilibrium of influences of ATP and CDC-37 on HSP-90 conformations changes dramatically when FKB-6 joins the complex. In the absence of ATP, the addition of FKB-6 increases the affinity of sB-Raf towards the chaperone machinery. Substantially less free sB-Raf is observed when FKB-6 is present (Fig. 8 step c). When ATP is added to sB-Raf•CDC-37•FKB-6•HSP-90 complexes, instead of complex disassociation, an increased sedimentation coefficient is observed in AUC, which implies that at this stage the HSP-90 machinery is performing the closing reaction and the closed complex then can continue the hydrolysis cycle well described for Hsp90 (see model Fig. 8 step d & step e). Hence the presence of FKB-6 shifts the balance towards the chaperon-bound and ATP-bound state (Fig. 8 step d) by generating a much more stable complex which is not likely to dissociate by the addition of ATP. This indicates that FKB-6 may be a strongly supportive factor in improving the efficiency of the HSP-90 ATPase cycle for kinases in addition to the own activity it may provide. This feature is shared mostly by Fkbp51 and Fkbp52.

### FKB-6 motifs are structurally associated at interaction hotspots in the complex interfaces

The stabilizing property of FKB-6 is further supported by the structural information of the kinase-chaperon complex obtained based on intermolecular crosslinked peptides. The comparison of intermolecular crosslinked pairs in the fully assembled complex imply that FKB-6 could directly strengthen the kinase-chaperon interface of the complex. Interestingly, the corresponding peptides of two reported hotspots in the kinase•Cdc37•Hsp90 structure (PDB: 5FWL^30,35^) are also detected as crosslinked pairs in our study. Free energy analysis of the binding interfaces in the human complex have been used to indicate regions as binding sites that mainly correspond to the residues AA110-140 and AA230-250 in Cdc37 and AA320-340 and AA400-420 in Hsp90^35^. The corresponding regions of CDC-37 (AA105-AA136) and HSP-90 (AA229-AA251) are clearly crosslinked in several variations. We previously reported the importance of CDC-37’s NTD for initial binding to HSP-90’s MD^12^, which is supposed to be the main interaction site for kinase binding. Due to conformational rearrangements necessary for kinase loading onto HSP-90^26,30,36^ an additional interaction of both middle and C-terminal domain of CDC-37 with HSP-90’s MD emerges that is also in agreement with one of the reported hotspots. The other site is the HSP-90•kinase interface of the sB-Raf N-lobe, where the protection of the flexible region in the kinase is achieved by interaction with HSP-90, which was also previously shown by us^9^. Here contributing HSP-90 regions were found to be crosslinked to sB-Raf, CDC-37 and FKB-6. This implies that FKB-6 could work on the client protein on similar sites in cooperation with HSP-90 and CDC-37. Attempts to dock the structure of FKB-6 onto the ternary complex based on the obtained crosslinks were not yielding conclusive results with fairly large distances remaining for the individual crosslinking sites (37.7 Å and 53.5 Å apart), which shows that a rigid docking of FKB-6 to the ternary complex may not reflect the flexibility of domains still remaining in HSP-90. It is reasonable to assume that a shift in a relative position of domains is induced by the presence of FKB-6, so that the whole complex is stabilized by inclusion of FKB-6. It is important to consider that the dimeric part of HSP-90 as well as the cofactors were aligned to the complex structure based upon the spacer length of the crosslinking reagent omitting orientations, where the linker length would be overstretched. Whilst assuming that only one of cofactors being present on the native mass spectrometry, this reduction might therefore pose a possible limitation of our approach.

Taken together our results demonstrate the contribution of FKB-6 to the kinase•HSP-90 chaperon complex and indicate the involvement of FKB-6 in the intermolecular interactions. They further reveal more information about the role of FKB-6 in the complex, which appears to guide the transfer of the kinase from CDC-37 to HSP-90 by stabilizing the quaternary complex with linking interactions between the individual components.

## Supplementary Information

Supplementary Information.

## Data Availability

All data are fully available without restriction.

## References

[1] Dey B, Lightbody JJ, Boschelli F (1996). CDC37 is required for p60v-src activity in yeast. Mol. Biol. Cell.

[2] Xu Y, Lindquist S (1993). Heat-shock protein hsp90 governs the activity of pp60v-src kinase. Proc. Natl. Acad. Sci. U S A.

[3] Dai K, Kobayashi R, Beach D (1996). Physical interaction of mammalian CDC37 with CDK4. J. Biol. Chem..

[4] Vaughan CK (2006). Structure of an Hsp90-Cdc37-Cdk4 complex. Mol. Cell.

[5] Holmes JL, Sharp SY, Hobbs S, Workman P (2008). Silencing of HSP90 cochaperone AHA1 expression decreases client protein activation and increases cellular sensitivity to the HSP90 inhibitor 17-allylamino-17-demethoxygeldanamycin. Cancer Res..

[6] Li J, Soroka J, Buchner J (1823). The Hsp90 chaperone machinery: Conformational dynamics and regulation by co-chaperones. Biochim. Biophys. Acta.

[7] Hessling M, Richter K, Buchner J (2009). Dissection of the ATP-induced conformational cycle of the molecular chaperone Hsp90. Nat. Struct. Mol. Biol..

[8] McLaughlin SH (2006). The co-chaperone p23 arrests the Hsp90 ATPase cycle to trap client proteins. J. Mol. Biol..

[9] Eckl JM, Daake M, Schwartz S, Richter K (2016). Nucleotide-free sB-Raf is preferentially bound by Hsp90 and Cdc37 in vitro. J. Mol. Biol..

[10] Taipale M (2012). Quantitative analysis of HSP90-client interactions reveals principles of substrate recognition. Cell.

[11] Basso AD (2002). Akt forms an intracellular complex with heat shock protein 90 (Hsp90) and Cdc37 and is destabilized by inhibitors of Hsp90 function. J. Biol. Chem..

[12] Eckl, J. M. *et al.* Hsp90.Cdc37 complexes with protein kinases form cooperatively with multiple distinct interaction sites. *J. Biol. Chem.* **290**, 30843–30854. 10.1074/jbc.M115.693150 (2015). 10.1074/jbc.M115.693150 PMC4692213 26511315

[13] Boczek EE (2015). Conformational processing of oncogenic v-Src kinase by the molecular chaperone Hsp90. Proc. Natl. Acad. Sci. U S A.

[14] Sinars CR (2003). Structure of the large FK506-binding protein FKBP51, an Hsp90-binding protein and a component of steroid receptor complexes. Proc. Natl. Acad. Sci. U S A.

[15] Kaziales A, Barkovits K, Marcus K, Richter K (2020). Glucocorticoid receptor complexes form cooperatively with the Hsp90 co-chaperones Pp5 and FKBPs. Sci. Rep..

[16] Vitt S (2020). Molecular and low-resolution structural characterization of the Na(+)-translocating glutaconyl-CoA decarboxylase from *Clostridium symbiosum*. Front. Microbiol..

[17] Peetz O (2019). LILBID and nESI: Different native mass spectrometry techniques as tools in structural biology. J. Am. Soc. Mass Spectrom..

[18] Morgner N, Robinson CV (2012). Massign: An assignment strategy for maximizing information from the mass spectra of heterogeneous protein assemblies. Anal. Chem..

[19] Plum S (2013). Combined enrichment of neuromelanin granules and synaptosomes from human substantia nigra pars compacta tissue for proteomic analysis. J. Proteomics.

[20] Barkovits, K. *et al.* Blood contamination in CSF and its impact on quantitative analysis of alpha-synuclein. *Cells* **9**. 10.3390/cells9020370 (2020). 10.3390/cells9020370 PMC7072133 32033488

[21] Tyanova S, Temu T, Cox J (2016). The MaxQuant computational platform for mass spectrometry-based shotgun proteomics. Nat. Protoc..

[22] Chen ZL (2019). A high-speed search engine pLink 2 with systematic evaluation for proteome-scale identification of cross-linked peptides. Nat. Commun..

[23] Combe CW, Fischer L, Rappsilber J (2015). xiNET: Cross-link network maps with residue resolution. Mol. Cell Proteomics.

[24] Eckl JM (2013). Cdc37 (cell division cycle 37) restricts Hsp90 (heat shock protein 90) motility by interaction with N-terminal and middle domain binding sites. J. Biol. Chem..

[25] Polier S (2013). ATP-competitive inhibitors block protein kinase recruitment to the Hsp90-Cdc37 system. Nat. Chem. Biol..

[26] Lotz GP, Lin H, Harst A, Obermann WM (2003). Aha1 binds to the middle domain of Hsp90, contributes to client protein activation, and stimulates the ATPase activity of the molecular chaperone. J. Biol. Chem..

[27] Wan PT (2004). Mechanism of activation of the RAF-ERK signaling pathway by oncogenic mutations of B-RAF. Cell.

[28] Haslbeck V (2015). The activity of protein phosphatase 5 towards native clients is modulated by the middle- and C-terminal domains of Hsp90. Sci. Rep..

[29] Richardson, J. M. *et al.* Cloning, expression and characterisation of FKB-6, the sole large TPR-containing immunophilin from *C. elegans*. *Biochem. Biophys. Res. Commun.* **360**, 566–572. 10.1016/j.bbrc.2007.06.080 (2007). 10.1016/j.bbrc.2007.06.080 17610845

[30] Verba KA (2016). Atomic structure of Hsp90-Cdc37-Cdk4 reveals that Hsp90 traps and stabilizes an unfolded kinase. Science.

[31] Morgner N, Kleinschroth T, Barth HD, Ludwig B, Brutschy B (2007). A novel approach to analyze membrane proteins by laser mass spectrometry: From protein subunits to the integral complex. J. Am. Soc. Mass Spectrom..

[32] Shaw AS, Kornev AP, Hu J, Ahuja LG, Taylor SS (2014). Kinases and pseudokinases: Lessons from RAF. Mol. Cell Biol..

[33] Nitika, Porter, C. M., Truman, A. W. & Truttmann, M. C. Post-translational modifications of Hsp70 family proteins: Expanding the chaperone code. *J. Biol. Chem.* **295**, 10689–10708. 10.1074/jbc.REV120.011666 (2020). 10.1074/jbc.REV120.011666 PMC7397107 32518165

[34] Backe SJ, Sager RA, Woodford MR, Makedon AM, Mollapour M (2020). Post-translational modifications of Hsp90 and translating the chaperone code. J. Biol. Chem..

[35] Czemeres J, Buse K, Verkhivker GM (2017). Atomistic simulations and network-based modeling of the Hsp90-Cdc37 chaperone binding with Cdk4 client protein: A mechanism of chaperoning kinase clients by exploiting weak spots of intrinsically dynamic kinase domains. PLoS ONE.

[36] Keramisanou D (2016). Molecular mechanism of protein kinase recognition and sorting by the Hsp90 kinome-specific cochaperone Cdc37. Mol. Cell.

