# Supplementary material for: HSP-90/kinase complexes are stabilized by the large PPIase FKB-6
Source: Sci Rep. 2021 Jun 11;11:12347. doi: 10.1038/s41598-021-91667-5 (PMC8196007; doi:10.1038/s41598-021-91667-5)
Supplement: Supplementary file 1 — Supplementary Information. [file 41598_2021_91667_MOESM1_ESM.pdf]

Supplemental figures

Supplemental Figure 1 Qualification of MS data analysis. Error distribution of all precusors in the (a) Q Exactive HF replicate and (b) the Q Exactive Plus replicate; Error distribution of filtered precursors in the (c) Q Exactive HF replicate and (d) in the Q Exactive Plus replicate; (e) Total amounts of spectra reported by the two analysis programms in different replicates. a stands for the results obtained from the analysis programm xMass; b for the results obtained from the analysis programm pLink. (f) Comparison of the amount of reported and unique peptide pairs in the MS analysis. Larger circle on the left side indicates results obtained from the xMass and small circle on the right side indicates reported results obtianed from the pLink.

a.

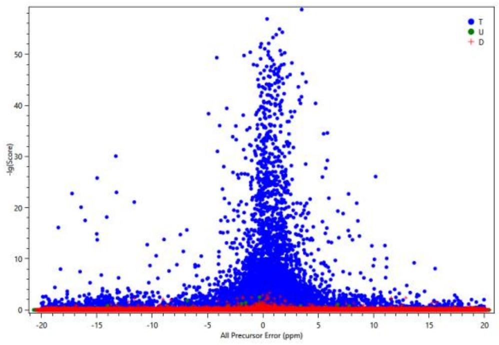

b.

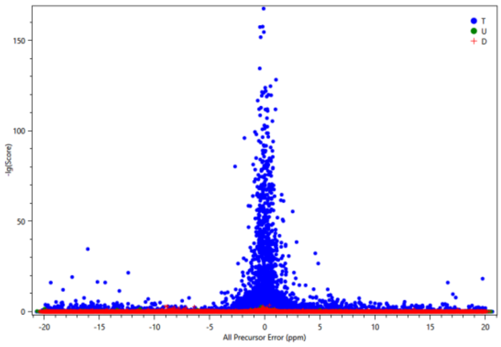

c.

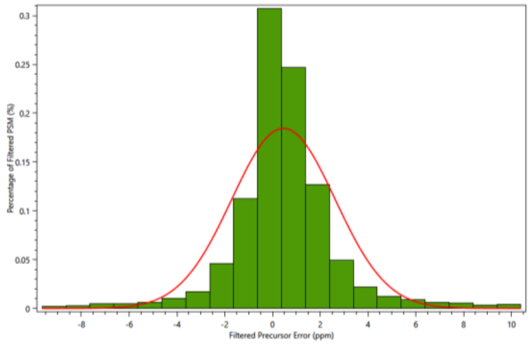

d.

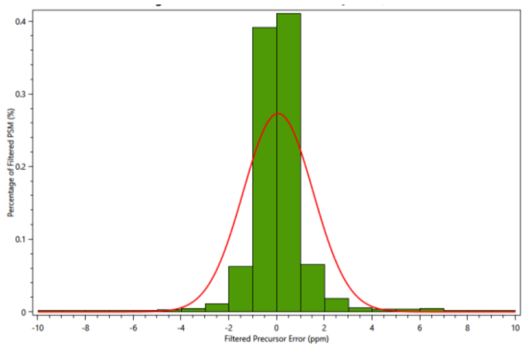

e.

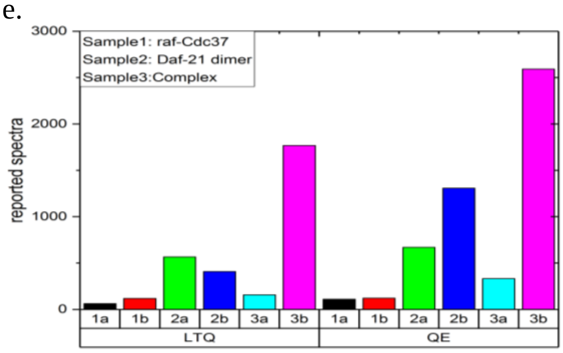

f.

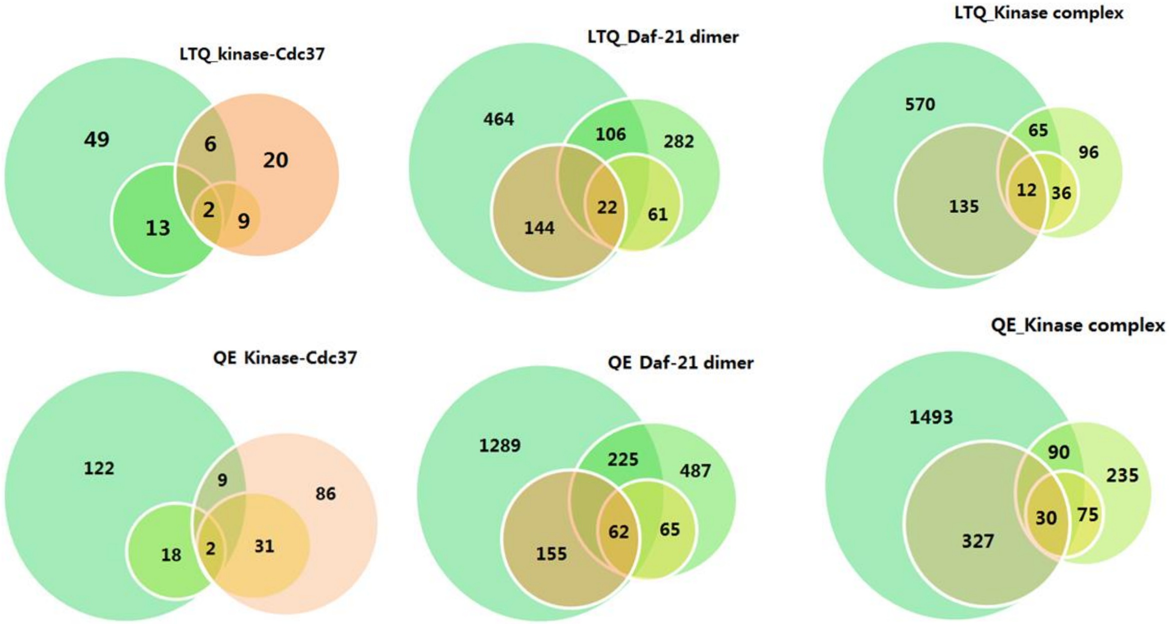
LTQ_kinase-Cdc37
49
6
20
13
2
9
LTQ_Daf-21 dimer
464
106
282
144
22
61
LTQ_Kinase complex
570
65
96
135
12
36
QE Kinase-Cdc37
122
9
86
18
2
31
QE Daf-21 dimer
1289
225
487
155
62
65
QE_Kinase complex
1493
90
235
327
30
75

Supplemental Figure 2 Influence of different nucleotides on the Raf•CDC-37•HSP-90 complex formation. (a) T = ATP; (b) D = ADP; (c) M = AMP-PNP; (d) rs = ATPγs. The order of added components is the same as the order written in the legend, which plays an important role in this case.

a.

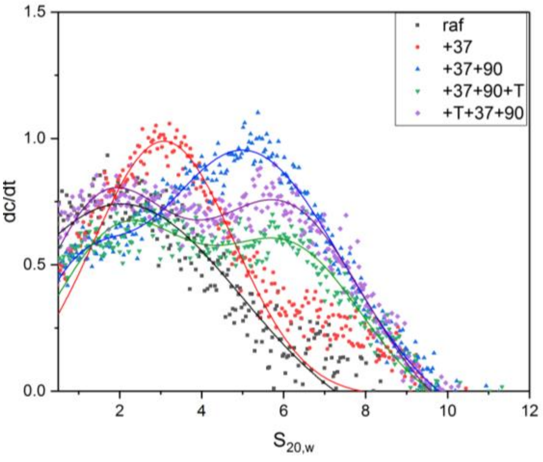

b.

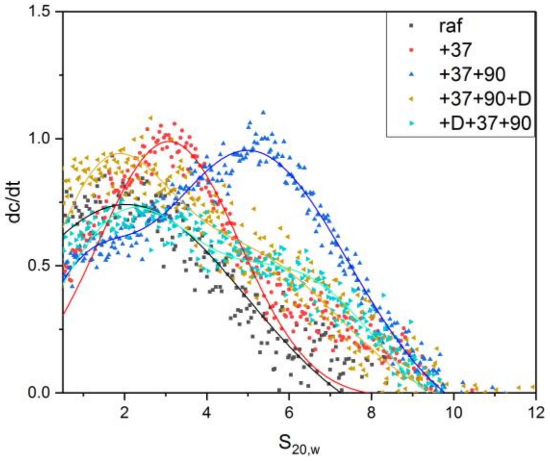

c.

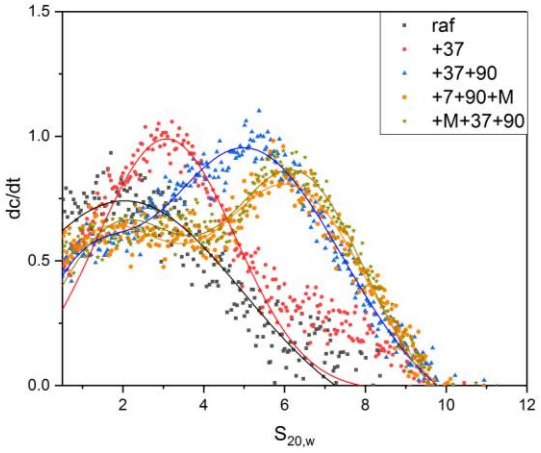

d.

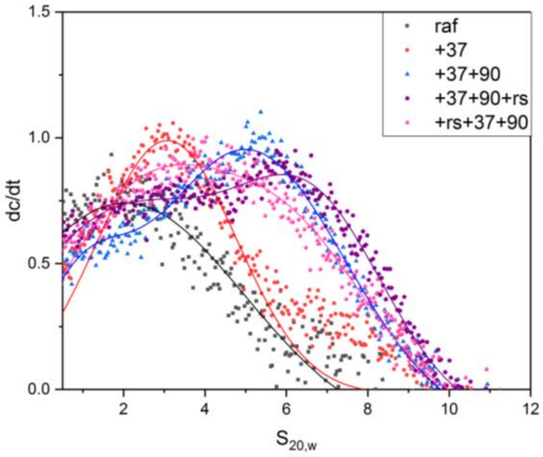

Supplemental Figure 3 Influence of nucleotides on the formation of Raf•CDC-37•HSP-90•FKB-6 complex. The letter A is short for the whole complex: Raf•·CDC-37•HSP-90•FKB-6. (a) Nucleotide used is ATP. (b) Nucleotide used is ADP. (c) Nucleotide used is AMP-PNP. (d) Nucleotide used is ATPγs.

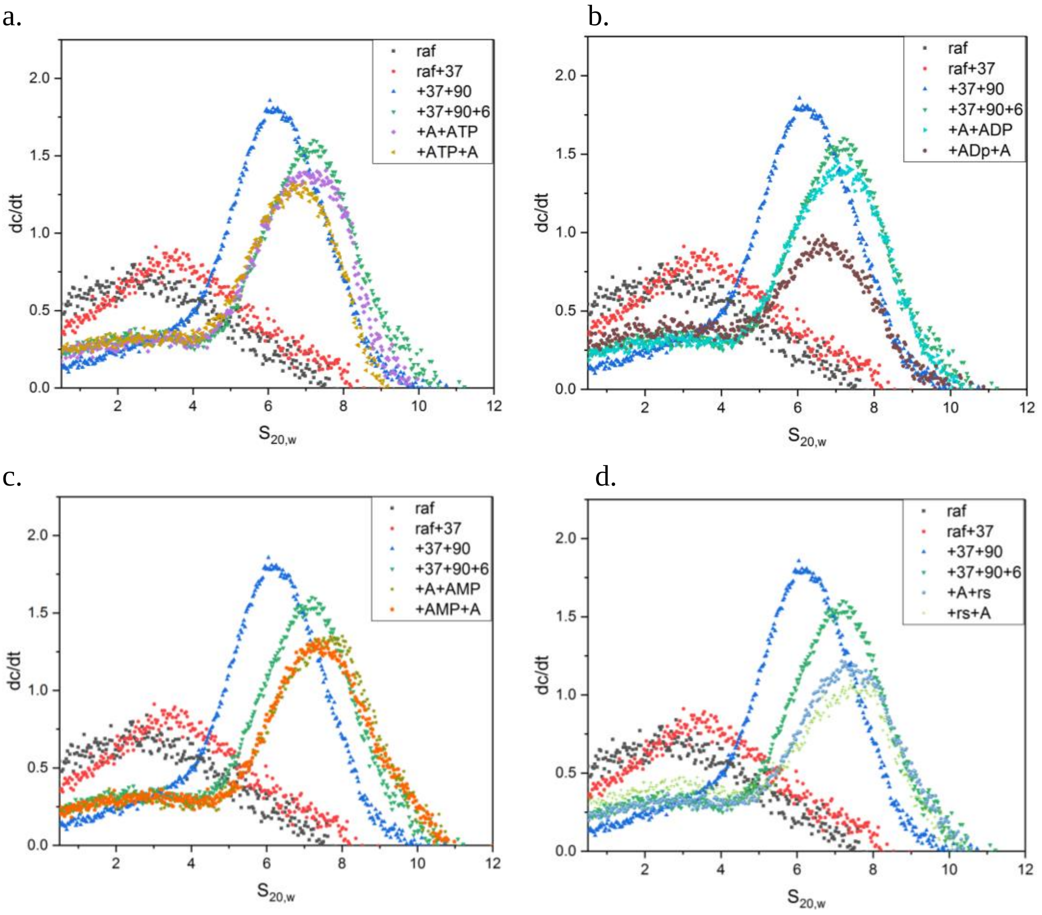

Supplemental Figure 4 Stoichiometry of protein components in the sB-Raf-chaperone complex. (a) Stepwise formation of the complex measured by UV absorbance at 280 nm. (b) Titration of the FKB-6 to sB-Raf•CDC-37•HSP-90 ternary complex.

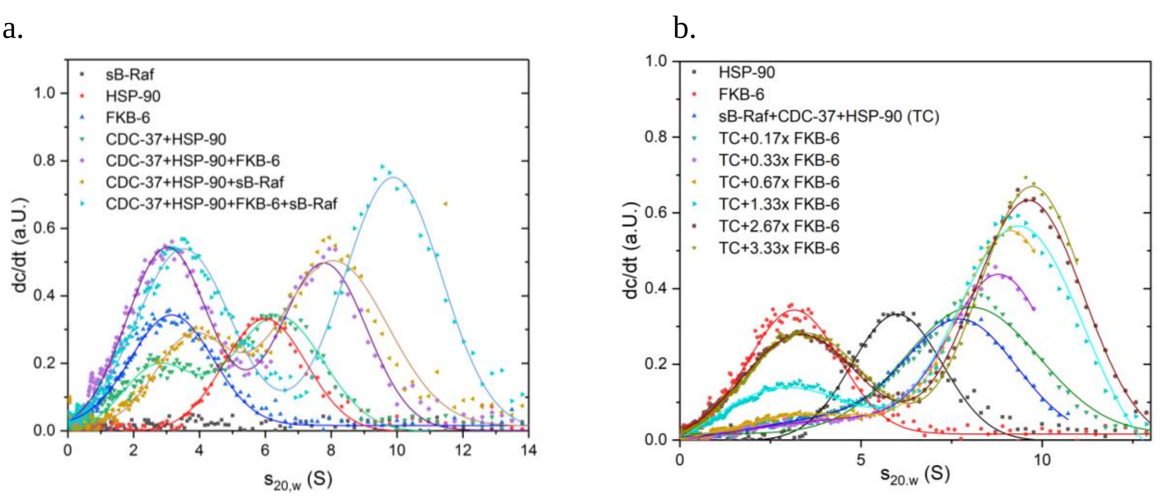

Supplemental Table 1 Most prominent unique hits in the crosslinked sample reported both by xMass and pLink in Q Exactive Plus and Q Exactive HF replicates. The blue colored part is obtained from the Q Exactive HF replicate while the white part is obtained from the Q Exactive Plus replicate. (A) Intermolecular crosslink pairs in the reference sample sb-Raf•CDC-37; (B) Both Inter- and intramolecular crosslinked pairs in the reference sample HSP-90 dimer; (C) Intermolecular crosslink pairs identified in the full complex sample.

A.

Peptide 1	Peptide 2	Protein 1	Protein 2
IGDFGLATVKSR	KPQAPK	sB-Raf (600)	CDC-37(165)
IGDFGLATVKSR	QFFKK	sB-Raf (600)	CDC-37(273)
IGDFGLATVKSR	KPQAPK	sB-Raf (600)	CDC-37(165)
IGDFGLATVKSR	QFFKK	sB-Raf (600)	CDC-37(273)

B.

Peptide 1	Peptide 2	Protein 1	Protein 2
APFDLFENKK	KNLVK	HSP-90(326)	HSP-90(385)
APFDLFENKK	NSIKLYVR	HSP-90(326)	HSP-90(333)
APFDLFENKKSK	SKNSIK	HSP-90(327)	HSP-90(329)
APFDLFENKK	KNLVK	HSP-90(326)	HSP-90(385)
APFDLFENKK	SKNSIK	HSP-90(326)	HSP-90(329)
ENQTQIYYITGESKDVVAASAFVER	IMKAQALR	HSP-90(469)	HSP-90(585)
FYEQFGKNLK	IMKAQALR	HSP-90(414)	HSP-90(585)
KFYEQFGK	IMKAQALR	HSP-90(407)	HSP-90(585)
MKENQTQIYYITGESK	EGLELPETEEEKK	HSP-90(455)	HSP-90(528)
YFEDEELNKTK	IKEIVK	HSP-90(263)	HSP-90(192)
YFEDEELNKTKPIWTR	KHSQFIGYPIK	HSP-90(263)	HSP-90(197)
YFEDEELNKTKPIWTR	NSIKLYVR	HSP-90(263)	HSP-90(333)

C.

Peptide 1	Peptide 2	Protein 1	Protein 2
EAELEEKER	KNLVK	CDC-37(137)	HSP-90(385)
ITEKKPQAPK	KFYEQFGK	CDC-37(164)	HSP-90(407)
SLNAVATNTTVQKQFFK	APFDLFENKK	CDC-37(269)	HSP-90(326)
QFFKK	SKNSIK	CDC-37(273)	HSP-90(329)
KFEAAEPVYMK	IGDFGLATVKSR	CDC-37(274)	sB-Raf(600)
KFEAAEPVYMK	APFDLFENKK	CDC-37(274)	HSP-90(326)
KFEAAEPVYMK	IGDFGLATVKSR	CDC-37(274)	sB-Raf(600)
KFEAAEPVYMKHYQDEVK	DSSTMGYMAAKK	CDC-37(284)	HSP-90(601)
FEAAEPVYMKHYQDEVK	GNVIKGWDLGVATMTK	CDC-37(284)	FKB-6(71)
FEAAEPVYMKHYQDEVK	IMKAQALR	CDC-37(284)	HSP-90(585)
GVAQKMDEEVFK	MKTLGR	CDC-37(359)	sB-Raf(22)
WKDIEVSDDEDDTHPNIDTPSLFR	KNLVK	CDC-37(42)	HSP-90(385)
MAEKKMEQEK	KNLVK	CDC-37(77)	HSP-90(385)

KMEQEK	ILKVIR	CDC-37(78)	HSP-90(381)
KMEQEK	SKNSIK	CDC-37(78)	HSP-90(329)
MEQEKIDKEK	NSIKLYVR	CDC-37(83)	HSP-90(333)
KMEELEK	EIVKK	CDC-37(94)	HSP-90(196)
RAEEVLEYEKSTDPEK	KPQAPK	FKB-6(288)	CDC-37(165)
RAEEVLEYEKSTDPEK	KFYEQFGK	FKB-6(288)	HSP-90(407)
KHSQFIGYPIK	KMEELEK	HSP-90(197)	CDC-37(94)
EKYFEDEELNK	KMEELEK	HSP-90(254)	CDC-37(94)
YFEDEELNKTKPIWTR	KLAAADVTDK	HSP-90(263)	CDC-37(101)
APFDLFENKK	QFFKK	HSP-90(326)	CDC-37(273)
APFDLFENKK	MAEKK	HSP-90(326)	CDC-37(77)
SKNSIK	MAEKK	HSP-90(329)	CDC-37(77)
NSIKLYVR	GTTSKK	HSP-90(333)	CDC-37(93)
NSIKLYVR	QFFKK	HSP-90(333)	CDC-37(273)
NSIKLYVR	IDKEK	HSP-90(333)	CDC-37(86)
NSIKLYVR	KMEELEK	HSP-90(333)	CDC-37(94)
NSIKLYVR	IDKEK	HSP-90(333)	CDC-37(86)
NSIKLYVR	KMEQEK	HSP-90(333)	CDC-37(78)
NSIKLYVR	QFFKK	HSP-90(333)	CDC-37(273)
KFYEQFGK	KPQAPK	HSP-90(407)	CDC-37(165)
KVEKVGVSNR	AQTKR	HSP-90(555)	CDC-37(304)
YQALTEPSELDTGKELFIK	KLAAADVTDK	HSP-90(62)	CDC-37(101)
YQALTEPSELDTGKELFIK	SDIQKQIDEVK	HSP-90(62)	CDC-37(115)
IGDFGLATVKSR	APFDLFENKK	sB-Raf(600)	HSP-90(326)
IGDFGLATVKSR	KMEQEK	sB-Raf(600)	CDC-37(78)
